# Synthesis, Acidity Constants and Tautomeric Structure of the Diazonium Coupling Products of 2-(Benzylsulfanyl)-7*H*-purin-6-one in Its Ground and Excited States

**DOI:** 10.3390/molecules16108788

**Published:** 2011-10-19

**Authors:** Elham S. Darwish, Mosselhi A. Mosselhi, Farag M. Altalbawy, Hosam A. Saad

**Affiliations:** 1 Department of Chemistry, Faculty of Science, University of Cairo, Giza, 12613, Egypt; 2 Department of Chemistry, Faculty of Science, University of Taif, Taif, 888, Saudi Arabia; Email: mosselhi2008@hotmail.com (M.A.M.); h_saadzag@yahoo.com (H.A.S.); 3 National Institute of Laser-enhanced Sciences, University of Cairo, Giza, 12613, Egypt; Email: f_altalbawy@yahoo.com

**Keywords:** purine, diazonium coupling, azo compounds, tautomerism, ground, excited states

## Abstract

A series of new 8-arylhydrazono-2-(benzylsulfanyl)-7*H*-purin-6-ones **6** were synthesized, their electronic absorption spectra in different organic solvents of varying polarities were investigated and their acid dissociation constants in both the ground and excited states were determined spectrophotometrically. The tautomeric structures of such products were elucidated by spectral analyses and correlation of their acid dissociation constants with the Hammett equation. The results indicated that the studied compounds **6** exist predominantly in the hydrazone tautomeric form **6A** in both the ground and excited states.

## 1. Introduction

Purines are ubiquitous nitrogen-containing heterocycles that exist at relatively high concentrations in living organisms [1]. Guanine and adenine, two of the most common purines, are essential components of nucleic acids (RNA and DNA), cofactors and signaling molecules that modulate protein function [2] and other fundamental biological processes [3,4,5,6]. Indeed, a great variety of di-, tri- or tetra-substituted purines described in the literature have been found to be potent inhibitors of chaperone HSP90, protein kinases (MAP, Src and Cdk), sulfotransferases, phosphodiesterases and microtubule assembly, inducers of interferon and dedifferentiation and antagonists of adenosine receptors and corticotropin-releasing hormone receptors [2]. This wide range of biological activities displayed by purines is conferred by a judicious choice of the nature of the substituents that can be combined on the N-1, C-2, N-3, C-6, N-7, C-8 and N-9 centers of purine moiety [4]. Furthermore, thiopurines are used as anti-cancer agents, in the treatment of inflammatory disorders, and as immunosuppressants [7]. Thiopurines are chemically more reactive than the normal DNA bases, so they have powerful therapeutic activity [8].

On the other hand, arylazo heterocycles are a versatile class of colored organic compounds that have recently attracted the interest of many research groups due to their diverse applications, not only as classical synthetic dyes and pigments, but also as solvatochromic probes and thermally stable organic second-order nonlinear optical (NLO) chromophores [9,10,11,12]. Other recent applications, include memory and recording devices, molecular switches, thermochromic, photovoltaic and fluorescent devices, supramolecular systems, holographic data storage materials, acid-base and metal sensors, active ligands in Pd-catalyzed cross-coupling reactions and lasers [10,13,14,15,16,17,18,19,20,21,22,23,24,25,26]. The biological importance of arylazo compounds is well known, and they are used as antineoplastics [27], anti-diabetics [28], antiseptics [29] and other useful chemotherapeutic agents [30,31,32,33].

Despite the fact that the purines, especially xanthine and their *N*-methyl-derivatives, are prone to easy electrophilic substitution reactions at the position 8 of the nucleus, it is to some extent surprising that only a few examples of 8-arylazopurines have been reported in the literature [34,35]. So it was considered worthwhile to study the reaction of arenediazonium salts with 2-(benzylsulfanyl)-7*H*-purin-6-one **5** to synthesize a series of 8-arylazo- analogues, **6**, and to determine their tautomeric structures prior to exploring their applications.

## 2. Results and Discussion

**Scheme 1:**
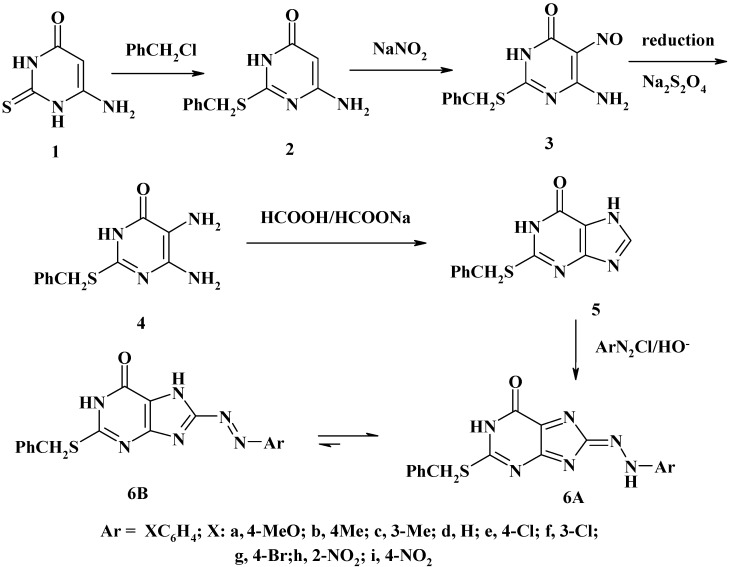
8-Arylhydrazono-2-benzylsulfanyl-1,8-dihydro-purin-6-ones.

The starting materials 6-amino-2-thiouracil (**1**) [36,37] and 6-amino-2-(benzylsulfanyl)uracil (**2**) [38,39] were prepared by literature methods. Treatment of **2** with an equivalent amount of sodium nitrite in dilute HCl afforded the new 5-nitrosouracil derivative **3**, which was reduced using sodium dithionite in water to give the corresponding 5,6-diamino-2-benzylsulfanyluracil (**4**). The ring closure of the latter was carried out by refluxing in a mixture of formic acid and sodium formate to afford 2-(benzylsulfanyl)-1,7-dihydropurin-6-one (**5**) [40,41]. The latter compound has proved to be versatile substrate in azo coupling reactions, allowing the preparation of several new donor acceptor substituted purines. The coupling reaction of arenediazonium salts with **5** in ethanol/sodium hydroxide gave rise to the formation of purine-azo dyes namely, the 8-arylhydrazono-2-benzylsulfanyl-1,8-dihydropurin-6-ones **6a-i**. Diazo coupling occurred selectively at the 8-position of the purine moiety to give compounds **6a-i** in moderate to good yields (35–60%), (Scheme 1). These results are in accordance with the selectivity of the reaction of electrophiles with xanthine and its *N*-methyl derivatives as it has been shown in the case of nitration reactions. [42] The structures of the formed 8-purine azo dyes **6a-i** were unambiguously confirmed by their analytical and spectral data (IR, ^1^H-NMR and MS).

The infrared spectra of dyes **6a-i** (see Experimental) showed the two characteristic bands at 3,100–3,172 and 3,300–3,397 cm^−1^ for two N-H stretching absorptions. The strong bands observed at 1,678–1,693 cm^−1^ and 1,595–1,653 cm^−1^ indicate stretchings vibrations of the C=O and C=N groups. Their ^1^H-NMR spectra in DMSO-d_6_ exhibited a broad singlet peak centered at δ 10.10–10.90 and 11.61–13.22 due to two NH protons. The peaks at δ 4.60–4.93 are characteristic for CH_2_ protons.

The ^13^C-NMR of **6a-i** in DMSO-d_6_ revealed peaks at δ 151.5–153.0 ppm which are characteristic for C8=N, hydrazo group and correspond with data for 8-substituted purine derivatives [43]. Typically, C8 is shifted downfield (by 13 ppm from 141 to 154 ppm) in comparison with 8-unsubstituted analogs [44,45].

Their mass spectra revealed in each case the respective molecular ion peak with low intensity. Although the foregoing spectroscopic data are consistent with the assigned structures **6a-i**, they cannot distinguish between the two possible tautomeric structures, namely, the arylazo and arylhydrazono forms **6A** and **6B**, respectively (Scheme 1). To elucidate the tautomeric structure of compounds **6**, we studied their electronic absorption spectra.

The electronic absorption spectra of compounds **6a-i** in dioxane revealed, in each case, two characteristic absorption bands in the regions 385–405 and 288–318 nm Table 1. The spectra of the unsubstituted compound **6d**, taken as representative example of the series prepared, in a series of different solvents, exhibit little λ_max_ shifts (Table 1). On the basis of such an absorption pattern, it can be concluded that the studied compounds **6** have in solution one tautomeric form, namely the hydrazone tautomer **6A**. This conclusion was confirmed by the ^1^H-NMR spectra of the studied compounds **6**. Such spectra showed the hydrazone NH proton signals in the region of δ 11.61–13.22 (see Experimental).

**Table 1:** Electronic absorption and spectral data of the compounds **6a-i**.

Compd. no.	λ_max_ nm (EtOH)	Compd. no.	λ_max_ nm (EtOH)
**6a**	387 (4.17), 315 (4.10)	**6f**	388 (4.38), 288 (4.52)
**6b**	390 (4.42), 310 (4.35)	**6g**	396 (4.50), 317 (4.47)
**6c**	385 (4.21), 295(4.05)	**6h**	395 (4.65), 318 (4.51)
**6d^ +^**	392 (4.26), 300 (4.45)	**6i**	405 (4.58), 310(4.33)
**6e**	394 (4.35), 298 (4.40)		

^+^ solvent: λ_max_ nm (Log ε) Ethanol: 398 (4.22), 300 (4.40); Chloroform: 388 (4.15), 315 (4.18); Acetic acid: 392(4.26), 312 (4.29); Cyclohexane: 405 (4.29), 305 (4.43); Pyridine: 410(4.12); 328 (4.15); Ether: 398 (4.04),310 (sh.).

**Figure 1:**
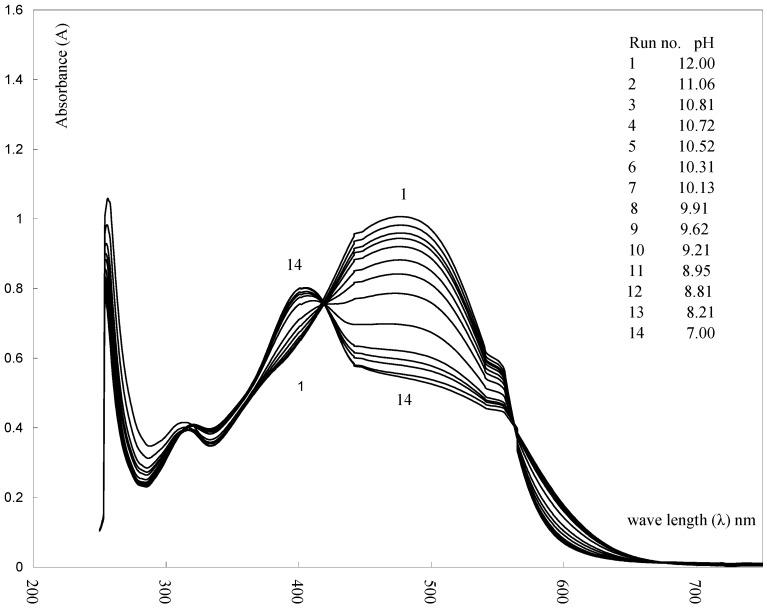
Electronic Absorption Spectra of (**6a**), in solution of different pH values (20% dioxane-water) at 27°C and μ=0.10.

**Figure 2:**
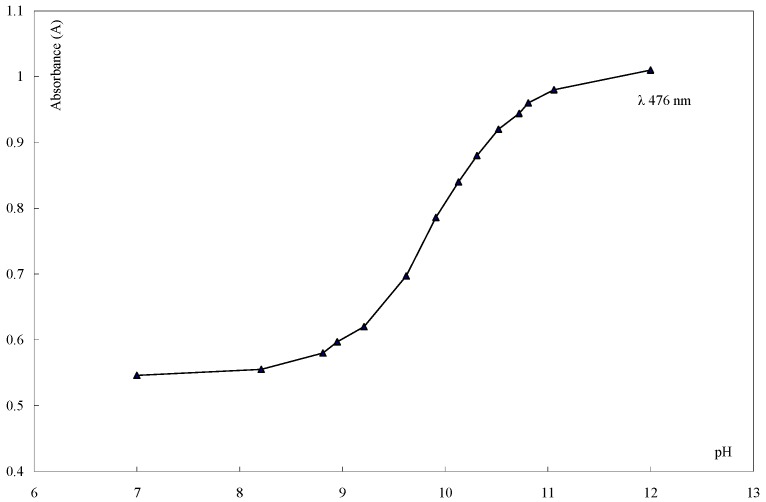
Spectrophotometric titration curve of (**6a**), at λ max.476 nm in 20% dioxane-water at 27°C and μ=0.10.

To provide further evidence for the tautomeric form **6A** assigned to the studied coupling products, the acid dissociation constants pKa of the series prepared were determined and their correlation by the Hammett equation was tested [46]. The pKa values for the series **6a-i** were determined spectrophotometrically at 27 °C in 80% dioxane-water mixture (v/v). In all determinations the ionic strength was kept constant at 0.1. From the pH-absorbance data. Typical absorption spectra of **6a** in such buffer solutions are shown in Figure 1 and Figure 2. The value of pKa was calculated (See experimental).

**Figure 3:**
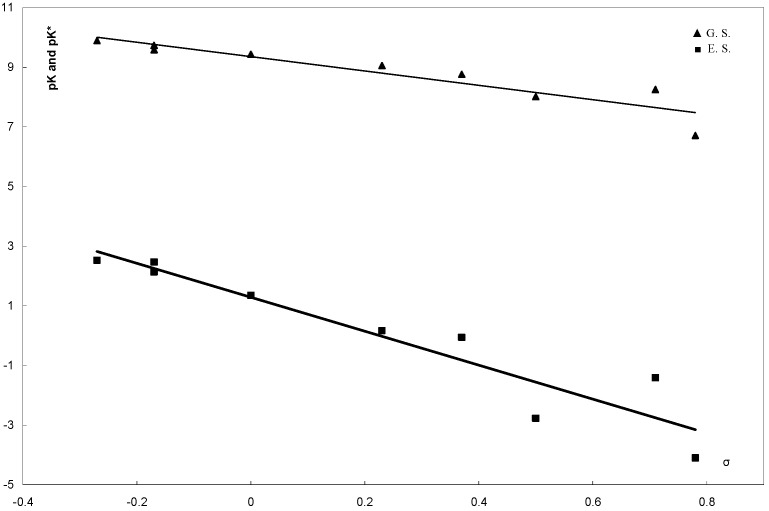
Correlation of pKa and pKa* 8-arylhydrazono-2-benzylsulfanyl-1,8-dihydro-purin-6-onee **6a-i** with the Hammett substituent constant σ_X_.

**Figure 4:**
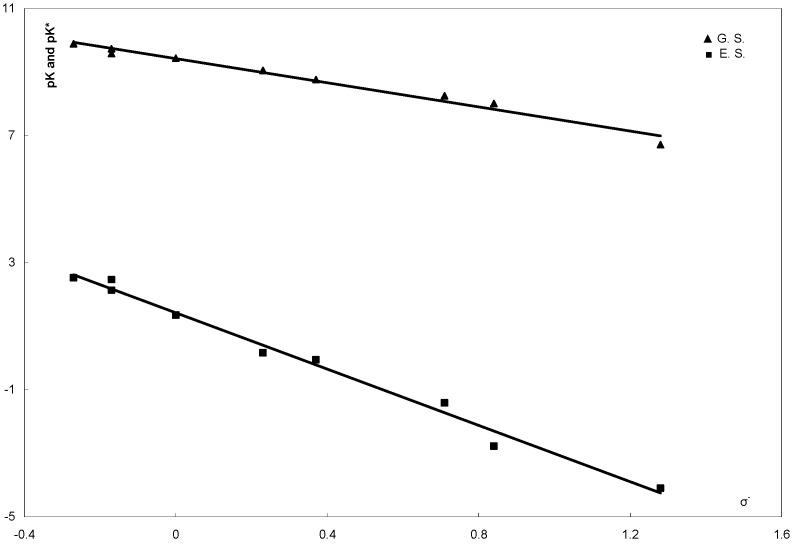
Correlation of pKa and pKa* 8-arylhydrazono-2-benzylsulfanyl-1,8-dihydro-purin-6-ones **6a-i** with the Hammett substituent constant σ^−^_X_.

The pKa values determined for the compounds **6a-i** are listed in Table 2. The pKa values were plotted *versus* the Hammett substituent constants σx and σ^−^x as shown in Figure 3 and Figure 4 [46].

**Table 2:** Acid dissociation constants pK and pK* of compounds **6a-i.**

Compd. No	σ	σ^−^	pK	λ max (a)	λ max (b)	∆ν cm^−1^	pK*
**6a**	−0.27	−0.27	9.89	405	476	3683	2.52
**6b**	−0.17	−0.17	9.73	408	479	3633	2.46
**6c**	−0.17	−0.17	9.58	400	470	3723	2.13
**6d**	0	0	9.43	402	480	4042	1.35
**6e**	0.23	0.23	9.05	397	482	4442	0.17
**6f**	0.37	0.37	8.76	405	493	4407	−0.05
**6g**	0.71	0.71	8.25	404	502	4832	−1.41
**6h**	0.78	1.28	6.71	415	535	5405	−4.10
**6i**	0.5	0.84	8.01	400	510	5392	−2.77

(a) in acid medium; (b) in alkaline medium; ± s = 0.04.

The equations corresponding to the straight lines obtained are:

pKa = 9.3598 − 2.4067σ_X_, r ^2^ = 0.8563, s = ± 0.160

**Scheme 2:**
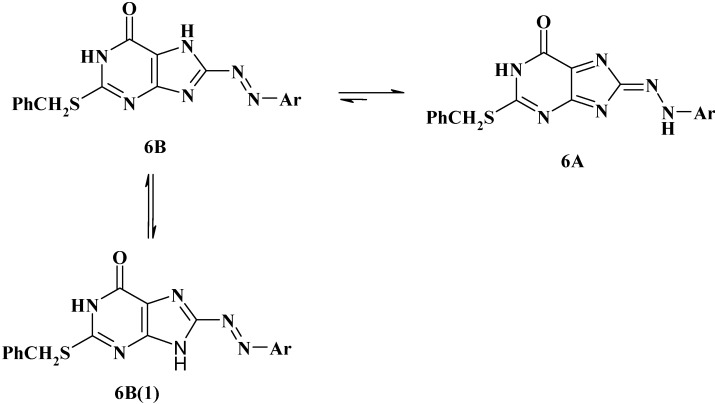
Azo –hydrazone tautomeric structures of compounds **6**.

pKa = 9.4255 − 1.8994 σ_X_^−^, r ^2^ = 0.977, s = ± 0.087

where r is the correlation coefficient and s is the standard deviation. From these values of r and s, the pKa data from **6a-i** seem to be better correlated with the enhanced Hammett substituent constant σ^-^x. This finding indicates that compounds **6a-i** exist in the hydrazone form **6A** in solution. This is because if **6** existed as equilibrium mixture of **6A** and **6B** (Scheme 1 and Scheme 2) no linear relations between pK and σ^−^x would be observed. Furthermore, the value of the reaction constant ρ = 1.8994 seems to favor the hydrazone form **6A** as it is in good agreement with those reported for similar hydrazones and not arylazo derivatives [47,48]. If the azo form **6B** were the predominant tautomer for the studied compounds **6**, the value of the reaction constant ρ would have been not more than 0.75. This is because the transmissive factor for the bridge -C=C-N=N- in the azo form **6B** is expected to be 0.32 as the transmissive factors of the -C=C- and -N=N- bridges were reported to be 0.47 and 0.69, respectively.

Next, the acid dissociation constants pK*'s of the studied compounds in excited state were calculated by utilizing the so-called Forester energy cycle [49,50]. According to this cycle:

pK* = pK + (Δν) (0.625/T)

where pK and pK* are the acid dissociation constants in the ground and excited states, respectively and Δν represents the frequency difference in cm^−1^ between the values of the absorption maximum λ_max_. of the compound in acid and alkaline media. The results of such calculations are summarized in Table 2. Correlation of these data of pK* with σx and σ^−^x are shown in Figure 3 and Figure 4, respectively. The linear equations corresponding to such correlations are:

pK* = 1.284 − 5.901σ_X_, r^2^ = 0.891, s = ± 0.139364

pK* = 1.4196 − 4.458σ_X_^−^, r^2 ^= 0.9884, s = ± 0.061538

Such linear equations indicate that studied compounds **6a-i** are predominantly in the hydrazone tautomeric form in their excited states. The larger value of ρ* emphasizes the importance of the electronic interaction in the excited state [51].

According to our further investigation, we have found another acidic proton in the products **6a-i**, it is measured as pKa of 1NH and it is found to be similar of, 10.72–10.80 for each derivative.


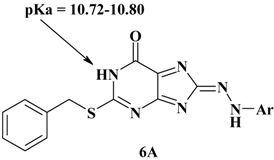


**Scheme 3:**
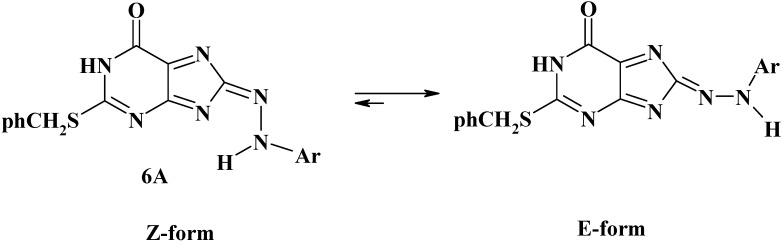
*E* and *Z* Forms of compounds **6**.

It is also found that the *E* isomer is energetically more stable than the *Z* isomer. Analogously, Compounds **6A** of our products **6a-i** can exist in the *E*-structure or the *Z*-structure (Scheme 3).

From the foregoing results, we can indicate that coupling of compound **5** with diazonium salts gives coupling products **6** having the hydrazone form **6A** in both the ground and excited states.

## 3. Experimental

### 3.1. General

All melting points were determined on an Electrothermal Gallenkamp apparatus and are uncorrected. The IR spectra were measured on a Pye-Unicam SP300 instrument in potassium bromide discs. The ^1^H-NMR spectra were recorded on a Varian Mercury VXR-300 spectrometer (300 MHz) and ^13^C-NMR was run at 75.46 MHz. The mass spectra were recorded on a GCMS-Q1000-EX Shimadzu and GCMS 5988-A HP spectrometers, the ionizing voltage was 70 eV. Electronic absorption spectra were recorded on Perkin-Elmer Lambda 40 spectrophotometer. Elemental analyses were carried out by the Microanalytical Center of Cairo University, Giza, Egypt. The starting materials 6-amino-2-thiouracil (**1**) [36,37] and 6-amino-2-benzylthiouracil (**2**) [38,39] were prepared as previously described.

*6-Amino-2-(benzylsulfanyl)-5-nitrosopyrimidin-4-one* (**3**): 6-Amino-2-(benzylsulfanyl)-pyrimidin-4-one (14.0 g, 0.06 mol) was suspended in an excess of dilute hydrochloric acid (3M, 100 mL) and a solution of sodium nitrite (5.0 g, 0.072 mol) in a little water was added gradually. The suspended material assumed a deep blue color and became crystalline and very bulky. The reaction was complete in about fifteen minutes, after which the solid material was filtered off, washed with cold water and then dried to give a blue powder; mp 198 °C; yield 86%; IR (KBr) ν/cm^−1^: 3,409 (NH), 3,319, 3,260 (NH_2_),1,647 (CO); ^1^H-NMR (DMSO-d_6_) δ/ppm: 4.45 (s, 2H, CH_2_), 5.60 (br s, 2H, NH_2_), 7.20–7.50 (m, 5H, Ar-H),10.80 (br s, 1H, NH); MS *m/z* (%): 299 (M^+^+1, 10.5), 298 (M^+^, 22.6), 111 (100.0), 92 (33.7), 83 (31.1), 64 (15.7); Anal. Calcd. for C_11_H_10_N_4_O_2_S (262.29): C, 50.37; H, 3.84; N, 21.36; S, 12.23%. Found: C, 50.22; H, 3.64; N, 21.20; S, 12.0%.

*5,6-Diamino-2-(benzylsulfanyl)* *pyrimidin-4-one* (**4**): 6-Amino-2-(benzylsulfanyl)-5-nitrosopyrimidin-4-one (**3**, 10 g, 40 mmol)) was suspended in distilled water (300 mL). The suspension was kept at 70–80 °C and sodium dithionite (ca. 18 g) was added upon stirring until the originally deeply colored solution had almost decolorized. After cooling to room temperature overnight, the solid formed was filtered, dissolved in dilute potassium hydroxide at 70 °C, filtered and cooled. The solution was acidified with glacial acetic acid and left to cool at room temperature overnight. The solid formed was filtered, washed with water, dried and stored in a vacuum desicator at room temperature to give pale yellow crystals; mp 244 °C; yield 70%; IR (KBr) ν/cm^–1^: 3,463 (NH), 3,350, 3,080 (NH_2_), 1,627 (CO); ^1^H-NMR (DMSO-d_6_) δ/ppm: 2.20 (br s, 2H, NH_2_), 4.51 (s, 2H, CH_2_), 5.50 (br s, 2H, NH_2_), 7.22–7.42 (m, 5H, Ar-H),10.45 (br s, 1H, NH); MS *m/z* (%): 248 (M^+^, 22.8), 215 (16.6), 157 (7.7), 126 (2.0), 91 (100), 65 (27.2), 55 (9.8); Anal. Calcd. for C_11_H_12_N_4_OS (248.3): C, 53.21; H, 4.87; N, 22.56; S, 12.91%. Found: C, 53.50; H, 4.58; N, 22.35 S, 13.0%.

*2-(Benzylsulfanyl)-7H-purin-6-one* (**5**) [40,41]: To a solution of *5,6-Diamino-2-(benzylsulfanyl)pyrimidin-4-one* (**4**, 5 g, 20 mmol) in formic acid (100 mL), an equivalent amount of sodium formate was added. The mixture was refluxed for 30 min. and the excess formic acid was distilled off *in vacuo*. The residue was suspended in distilled water, collected by filtration, washed with water, and dried. to afford the title compound **5** as colorless crystals; mp = 260 °C [from a mixture of dimethylformamide and water (v/v 1:2)]; yield 75%; IR (KBr) ν/cm^−1^: 3,338, 3,112 (2 NH), 1,660 (CO); ^1^H-NMR (DMSO-*d_6_*) δ/ppm: 4.60 (s, 2H, CH_2_), 7.34−7.91 (m, 5H, Ar-H), 8.19 (s, 1H, 8-CH), 10.51 (br s, 1H, NH), 12.24 (br s, 1H, NH); MS *m/z* (%): 258 (M^+^, 1.5), 151 (96.7), 134 (12), 123 (7.6), 91 (16.4), 69 (36.4), 55 (74.5), 54 (100); Anal. Calcd. for C_12_H_10_N_4_OS (258.3): C, 55.80; H, 3.90; N, 21.69; S, 12.41%. Found: C, 55.85; H, 3.80; N, 21.53; S, 12.32%.

### 3.2. General Procedure for Synthesis of 8-Arylhydrazono-2-benzylsulfanyl-1,8-dihydro-purin-6-ones **6a-i**

To a stirred solution of compound **5** (0.65 g, 2.5 mmol) in ethanol (20 mL) was added sodium hydroxide (0.1 g, 2.5 mmol) and the mixture was cooled in an ice bath to 0–5 °C. To the resulting solution, while being stirred, was added dropwise over a period of 20 min a solution of the appropriate arenediazonium chloride, prepared as usual by diazotizing the respective aniline (2.5 mmol) in hydrochloric acid (6 M, 1.5 mL) with sodium nitrite (1 M, 2.5 mL). The whole mixture was then left in a refrigerator overnight. The precipitated solid was filtered, washed with water and finally crystallized from dimethylformamide/ethanol (v:v 1:5) to give the respective hydrazones **6**. The physical constants and the spectral data of the products (**6a-i**) are listed below.

*2-(Benzylsulfanyl)-8-[(4-methoxyphenyl)hydrazono]-1,8-dihydropurin-6-one* (**6a**). Yellow solid; mp > 300 °C; yield 66%; IR (KBr) ν/cm^−1^: 3,303, 3,128 (NH), 1,685, 1,648 (CO); ^1^H-NMR (DMSO-*d_6_*) δ/ppm: 3.40 (s, 3H, OCH_3_), 4.70 (s, 2H, CH_2_), 7.01–7.40 (m, 4H, Ar-H), 7.60–7.92 (m, 5H, Ar-H), 10.84 (br s, 1H, NH), 13.05 (br s, 1H, NH); ^13^C-NMR (DMSO-*d_6_*): 34.8 (CH_2_), 51.0 (OCH_3_), [Aromatic-C, 118.3, 120.2-133.8], 151.5 (C8=N, hydrazo), 152.7 (C6), 155.3 (C4), 156.1 (C2), 158.6 (C5); MS *m/z* (%): 392 (M^+^, 4.3), 281 (44.8), 258 (20.7), 123 (20.7), 119 (27.6), 108 (100), 64 (48.3); Anal. Calcd. for C_19_H_16_N_6_O_2_S (392.43): C, 58.15; H, 4.11; N, 21.42; S, 8.17%. Found: C, 58.22; H, 4.18; N, 21.31; S, 8.25%.

*2-(Benzylsulfanyl)-8-[(4-methylphenyl)hydrazono]-1,8-dihydropurin-6-one* (**6b**). Yellow solid; mp > 300 °C; yield 71%; IR (KBr) ν/cm^−1^: 3,313, 3,133 (NH), 1,686, 1,640 (CO); ^1^H-NMR (DMSO-*d_6_*) δ/ppm: 2.34 (s, 3H, CH_3_), 4.60 (s, 2H, CH_2_), 7.02–7.35 (m, 4H, Ar-H), 7.50–7.90 (m, 5H, Ar-H), 10.76 (br s, 1H, NH), 12.93 (br s, 1H, NH); ^13^C-NMR (DMSO-*d_6_*): 24.5 (CH_3_), 34.9 (CH_2_), [Aromatic-C, 116.2, 121.5–136.0], 152.4 (C8=N, hydrazo), 153.0 (C6), 155.5 (C4), 156.7 (C2), 159.3 (C5); MS *m/z* (%): 376 (M^+^, 1.8), 272 (26.9), 226 (15.9), 139 (15.9), 122 (15.3), 108 (20.7), 92 (23.4), 77 (13.8), 65 (48.9), 60 (100); Anal. Calcd. for C_19_H_16_N_6_OS (376.43): C, 60.62; H, 4.28; N, 22.33; S, 8.52%. Found: C, 60.60; H, 4.15; N, 22.25; S, 8.60%.

*2-(Benzylsulfanyl)-8-[(3-methylphenyl)hydrazono]-1,8-dihydropurin-6-one* (**6c**). Yellow solid; mp > 300 °C; yield 68%; IR (KBr) ν/cm^−1^: 3,397, 3,127 (NH), 1,683, 1,645 (CO); ^1^H-NMR (DMSO-*d_6_*) δ/ppm: 2.23 (s, 3H, CH_3_), 4.50 (s, 2H, CH_2_), 7.30–7.85 (m, 9H, Ar-H), 10.41 (br s, 1H, NH), 12.72 (br s, 1H, NH); ^13^C-NMR (DMSO-*d_6_*): 25.5 (CH_3_), 34.0 (CH_2_), [Aromatic-C, 118.2, 120.0-134.6], 152.0 (C8=N, hydrazo), 152.9 (C6), 155.0 (C4), 156.5 (C2), 159.0 (C5); MS *m/z* (%): 376 (M^+^, 2.6), 270 (25.0), 149 (8.4), 123 (6.3), 91 (6.9), 77 (100); Anal. Calcd. for C_19_H_16_N_6_OS (376.43): C, 60.62; H, 4.28; N, 22.33; S, 8.52%. Found: C, 60.60; H, 4.15; N, 22.25; S, 8.60%.

*2-(Benzylsulfanyl)-8-[(phenylhydrazono]-1,8-dihydropurin-6-one* (**6d**). Yellow solid; mp > 300 °C; yield 60%; IR (KBr) ν/cm^−1^: 3,307, 3,133 (NH), 1,687, 1,644 (CO); ^1^H-NMR (DMSO-*d_6_*) δ/ppm: 4.70 (s, 2H, CH_2_), 7.250–7.86 (m, 10H, Ar-H), 10.80 (br s, 1H, NH), 13.10 (br s, 1H, NH); ^13^C-NMR (DMSO-*d_6_*): 33.0 (CH_2_), [Aromatic-C, 120.8, 122-129.6, 130.7, 131.0, 131.7, 137.5], 152.2 (C8=N, hydrazo), 152.8 (C6), 155.0 (C4), 157.0 (C2), 159.9 (C5); MS *m/z* (%): 362 (M^+^, 4.9), 255 (6.4), 227 (10.6), 167 (14.8), 130 (6.4), 123 (5.8), 111(10.1), 104 (14.5), 93 (19.9), 77 (100), 65 (17.6); Anal. Calcd. for C_18_H_14_N_6_OS (362.41): C, 59.65; H, 3.89; N, 23.19; S, 8.85%. Found: C, 59.60; H, 4.0; N, 23.10; S, 8.73%.

*2-(Benzylsulfanyl)-8-[(4-chlorophenyl)hydrazono]-1,8-dihydropurin-6-one* (**6e**). Yellow solid; mp > 300 °C; yield 83%; IR (KBr) ν/cm^−1^: 3,300, 3,126 (NH), 1,692, 1,653 (CO); ^1^H-NMR (DMSO-*d_6_*) δ/ppm: 4.80 (s, 2H, CH_2_), 7.10–7.25 (m, 4H, Ar-H), 7.35–7.89 (m, 5H, Ar-H), 10.84 (br s, 1H, NH), 13.05 (br s, 1H, NH); ^13^C-NMR (DMSO-*d_6_*): 33.0 (CH_2_), [Aromatic-C, 118.2, 120.8, 122.5–129.6, 130.6, 131.0, 131.7, 138.5], 153.0 (C8=N, hydrazo), 153.8 (C6), 155.8 (C4), 156.5 (C2), 159.0 (C5); MS *m/z* (%): 397 (M^+^+1, 8.1), 396 (M^+^, 5.5), 250 (6.4), 132 (10), 111(100), 75 (83.3), 69 (76.7), 55 (45); Anal. Calcd. for C_18_H_13_ClN_6_OS (396.85): C, 54.48; H, 3.30; N, 21.18; S, 8.08%. Found: C, 54.45; H, 3.30; N, 21.12; S, 8.0%.

*2-(Benzylsulfanyl)-8-[(3-chlorophenyl)hydrazono]-1,8-dihydropurin-6-one* (**6f**). Yellow solid; mp > 300 °C; yield 73%; IR (KBr) ν/cm^−1^: 3,321, 3,100 (NH), 1,685, 1,621 (CO); ^1^H-NMR (DMSO-*d_6_*) δ/ppm: 4.90 (s, 2H, CH_2_), 7.30–7.85 (m, 9H, Ar-H)), 10.10 (br s, 1H, NH), 12.18 (br s, 1H, NH); ^13^C-NMR (DMSO-*d_6_*): 33.0 (CH_2_), [Aromatic-C, 119, 122.0–129.5, 130.0, 132.8, 133.0, 138.0], 151.8 (C8=N, hydrazo), 152.3 (C6), 154.5 (C4), 155.9 (C2), 158.5 (C5); MS *m/z* (%): 397 (M^+^ + 1, 10.2), 396 (M^+^, 6.5), 255 (6.1), 132 (15), 111(100), 75(80.0), 69 (50.5), 55 (40); Anal. Calcd. for C_18_H_13_ClN_6_OS (396.85): C, 54.48; H, 3.30; N, 21.18; S, 8.08%. Found: C, 54.22; H, 3.50; N, 21.0; S, 8.20%.

*2-(Benzylsulfanyl)-8-[(4-bromophenyl)hydrazono]-1,8-dihydropurin-6-one* (**6g**). Orange solid; mp > 300 °C; yield 70%; IR (KBr) ν/cm^−1^: 3,323, 3,144 (NH), 1,688, 1,638 (CO); ^1^H-NMR (DMSO-*d_6_*) δ/ppm: 4.72 (s, 2H, CH_2_), 7.20–7.35 (m, 4H, Ar-H), 7.45–7.95 (m, 5H, Ar-H), 10.87 (br s, 1H, NH), 11.61 (br s, 1H, NH); ^13^C-NMR (DMSO-*d_6_*): 33.0 (CH_2_), [Aromatic-C, 120.0, 122.5-129.0, 130.6, 131.0, 131.7, 138.5], 152.5 (C8=N, hydrazo), 153.2 (C6), 155.0 (C4), 155.9 (C2), 158.7 (C5); MS *m/z* (%): 441 (M^+^, 8.6), 191 (77.2), 151 (100), 91 (77.2), 65 (86.3); Anal. Calcd. for C_18_H_13_BrN_6_OS (441.3): C, 48.99; H, 2.97; N, 19.04; S, 7.27%. Found: C, 48.90; H, 3.0; N, 19.15; S, 7.20%.

*2-(Benzylsulfanyl)-8-[(2-nitrophenyl)hydrazono]-1,8-dihydropurin-6-one* (**6h**). Orange solid; mp > 300 °C; yield 58%; IR (KBr) ν/cm^−1^: 3,385, 3,125 (NH), 1,693, 1,649 (CO); ^1^H-NMR (DMSO-*d_6_*) δ/ppm: 4.87 (s, 2H, CH_2_), 7.50–7.95 (m, 9H, Ar-H), 10.90 (br s, 1H, NH), 13.19 (br s, 1H, NH); ^13^C-NMR (DMSO-*d_6_*): 34.0 (CH_2_), [Aromatic-C, 120.0, 122.5–129.5, 130.0, 132.8, 133.0, 137.5], 152.0 (C8=N, hydrazo), 152.5 (C6), 153.8 (C4), 155.0 (C2), 158.0 (C5); MS *m/z* (%): 407 (M^+^, 18.8), 149 (45.9), 91 (27.5), 83 (34.6), 77 (26), 69 (52); Anal. Calcd. for C_18_H_13_N_7_O_3_S (407.41): C, 53.07; H, 3.22; N, 24.07; S, 7.87%. Found: C, 53.0; H, 3.30; N, 23.99; S, 7.80%.

*2-(Benzylsulfanyl)-8-[(4-nitrophenyl)hydrazono]-1,8-dihydropurin-6-one* (**6i**). Orange solid; mp > 300 °C; yield 66%; IR (KBr) ν/cm^−1^: 3,387, 3,127 (NH), 1,696, 1,651 (CO); ^1^H-NMR (DMSO-*d_6_*) δ/ppm: 4.93 (s, 2H, CH_2_), 7.15–7.25 (m, 4H, Ar-H), 7.40–7.88 (m, 5H, Ar-H), 10.94 (br s, 1H, NH), 13.22 (br s, 1H, NH); ^13^C-NMR (DMSO-*d_6_*): 35.0 (CH_2_), [Aromatic-C, 120.0, 122.5–129.0, 130.6, 131.0, 131.7, 138.5], 152.0 (C8=N, hydrazo), 152.9 (C6), 155.5 (C4), 156.9 (C2), 159.0 (C5); MS *m/z* (%): 407 (M^+^, 20), 150 (17.5), 97 (29.2), 83 (32.6), 77 (100), 68 (18); Anal. Calcd. for C_18_H_13_N_7_O_3_S (407.41): C, 53.07; H, 3.22; N, 24.07; S, 7.87%. Found: C, 53.0; H, 3.30; N, 23.99; S, 7.80%.

### 3.3. pK Determination

The acid dissociation constants pK's of the compounds **6** were determined spectrophotometrically in 80% (v/v) dioxane-water mixture at 27 °C and ionic strength 0.1. An Orion 420A pH meter fitted with combined glass electrode type 518635 was employed for measurement of pH values. The instrument was accurate to ±0.01 pH unit. It was calibrated using two standard Beckman buffer solutions of pH 4.01 and 7.00. The pH meter readings (B) recorded in dioxane-water solutions were converted to hydrogen ion concentration [H^+^] by means of the widely used relation of van Uitert and Haas [49] namely:

−log [H^+^] = B + log U_H_

where log U_H_ is the correction factor for the solvent composition and ionic strength used for which B is read. The value of log U_H_ was determined by recording the pH values for a series of hydrochloric acid and sodium chloride such that the ionic strength is 0.1 in 4:1 (v/v) dioxane-water mixture at 27 °C. The value of log U_H_ was found to be −0.48.

The experimental procedure followed in the determination of pka constants and their calculations from the absorbance-pH data are as previously described [50]. The pKa values were reproducible to ±0.04 pKa unit. The results are given in Table 2.

## 4. Conclusions

In conclusion, 2-(benzylsulfanyl)-7*H*-purin-6-one (**5**) [40,41] is prepared. A simple coupling reaction of **5** for the preparation of 8-arylhydrazono-2-benzylsulfanyl-1,8-dihydropurin-6-ones **6a-i** in good yield is described. The structures of the newly synthesized compounds **6a-i** were confirmed by spectral and elemental analyses data and the correlation with Hammett equation. The obtained results indicate that the isolated coupling products **6** have the hydrazone form **6A **in both the ground and excited states.
